# The effect of PFAS exposure on glucolipid metabolism in children and adolescents: a meta-analysis

**DOI:** 10.3389/fendo.2024.1261008

**Published:** 2024-02-14

**Authors:** Qingqing Zheng, Wu Yan, Shenghu Gao, Xiaonan Li

**Affiliations:** ^1^ Department of Children Health Care, Children’s Hospital of Nanjing Medical University, Nanjing, China; ^2^ Institute of Pediatric Research, Nanjing Medical University, Nanjing, China

**Keywords:** per- and polyfluoroalkyl substances, PFAS, glucolipid metabolism, glycometabolism, lipid metabolism, children, adolescents, meta-analysis

## Abstract

**Background:**

Previous studies showed that per- and polyfluoroalkyl substances (PFAS), which are widely found in the environment, can disrupt endocrine homeostasis when they enter the human body. This meta-analysis aimed to evaluate current human epidemiological evidence on the relationship between PFAS exposure and glucolipid metabolism in childhood and adolescence.

**Methods:**

We searched PubMed, Web of Science, Embase, and Cochrane Library databases, and identified population-based epidemiological studies related to PFAS and glucolipid metabolism indexes that were published before 30 December 2022. The heterogeneity of the included literature was assessed using the I-square (*I^2^*) test and statistics *Q*. Random-effects and fixed-effects models were used to combine the effect size. Subgroup analysis based on age and sex of the study participants was performed. A sensitivity analysis was used to evaluate the robustness and reliability of the combined results. Egger’s and Begg’s tests were used to analyze publication bias.

**Results:**

A total of 12 studies were included in this analysis. There was a positive association between PFAS and TC (*β* = 1.110, 95% CI: 0.601, 1.610) and LDL (*β* = 1.900, 95% CI: 1.030, 2.770), and a negative association between PFAS and HOMA-IR in children and adolescents (*β* = −0.130, 95% CI: −0. 200, −0.059). PFOS was significant positive associated with TC (*β* = 8.22, 95% CI: 3.93, 12.51), LDL (*β* = (12.04, 95% CI: 5.08, 18.99), and HOMA-IR (*β* = −0.165, 95% CI: −0.292, −0.038). Subgroup analysis showed that exposure to PFAS in the adolescent group was positively associated with TC and LDL levels, and the relationship was stronger in females.

**Conclusion:**

PFAS exposure is associated with glucolipid metabolism in children and adolescents. Among them, PFOS may play an important role. Recognition of environmental PFAS exposure is critical for stabilizing the glycolipid metabolism relationship during the growth and development of children and adolescents.

## Introduction

Chronic non-communicable diseases, mainly cardiovascular diseases, cancer, chronic respiratory diseases, and diabetes, are the leading causes of death worldwide (1). Overweight/obesity and abnormal glucolipid metabolism are major risk factors for these diseases, with a trend towards younger age, prevalent in childhood and adolescence, causing damage that may affect adult lives. Increased exposure to environmental pollutants may lead to harmful metabolic abnormalities, which may increase the incidence of chronic diseases (2). Therefore, some scholars have proposed the concept of Environmental Endocrine Disruptors (EEDs), a class of exogenous chemicals commonly found in the environment that can interfere with endocrine homeostasis and adversely affect the metabolism and other functions of the organism after entering the body (3).

Per- and polyfluoroalkyl substances (PFAS), are common synthetic organic compounds with outstanding thermal stability, chemical stability, hydrophobic oiliness, and high surface activity owing to their high-energy carbon–fluorine covalent bonds (4). They are widely used in commercial, industrial, and other fields, such as food packaging materials, carpets, shampoo, leather, paint, fire foam, waterproof and anti-fouling cleaning goods, and other household products containing PFAS substances (5, 6). Currently, more than 9,000 PFAS have been registered by the US Environmental Protection Agency (7).

Humans are widely exposed to PFAS directly or indirectly through air, soil, diet, drinking water, and food packaging. Moreover, owing to their chemical stability, PFAS are difficult to degrade in natural environments, which may eventually result in persistent pollutants. At the same time, the half-life of most PFAS can also range from months to years, which in turn bioaccumulates in the body after long-term exposure to the environment. As the most widely studied PFAS, perfluorooctanoic acid (PFOA), perfluorooctane sulfonic acid (PFOS), and perfluorohexane sulfonic acid (PFHxS) are commonly detected in human serum (8). Toxicological studies have shown that PFAS have a wide range of toxic effects, such as liver toxicity, kidney toxicity, neurotoxicity, endocrine toxicity, immunotoxicity, reproductive and developmental toxicity, and potential carcinogenicity (9–11), including humans. Although many countries and even coalition organizations have adopted policies to regulate the use of PFAS, long-term exposure would affect glucolipid metabolism since they remain in the body for months or even years (12). Therefore, endocrine toxicity of PFAS has attracted global attention.

Epidemiological studies based on populations have shown that doubling concentrations of PFOS and PFOA are associated with higher homeostasis model assessment of insulin resistance (HOMA-IR) and glycated hemoglobin (HbA1c) (13). However, contrary results have also suggested that long-term exposure to PFAS may be associated with lower insulin resistance in adults (14). In terms of lipid metabolism, studies have shown that PFAS exposure is associated with higher levels of low-density lipoprotein (LDL), high-density lipoprotein (HDL), and total cholesterol (TC) (15). Currently, most studies on PFAS exposure have focused on adults, especially pregnant women, whereas only a few studies have mentioned children. It has been shown that children are exposed to PFAS by similar routes as adults, but the exposure from hand to mouth via carpets is greater than that of adults. PFOA and PFOS in children under 12 years of age were estimated to be 101–219 ng/kg/day and 65.2–128 ng/kg/day, respectively, under high exposure scenarios (16). Nevertheless, children have unique physiological structures and behavioral characteristics during the growth and development period, which make them more prone to exposure to various environmental pollutants and more vulnerable to disease consequences than adults (17). An increasing number of studies have focused on the endocrine-disrupting effects of PFAS in children, but the conclusions have been inconsistent.

Therefore, it is necessary to seek strong evidence to explain the relationship between PFAS exposure and glucolipid metabolism in children and adolescents. We conducted a meta-analysis to clarify the relationship between PFAS exposure and glucolipid metabolism in children and adolescents and to provide a scientific theoretical basis for the health risk assessment of PFAS exposure and children’s growth and development.

## Materials and methods

### Search methods

We searched for relevant papers in PubMed, Web of Science, Embase, and Cochrane Library databases, which were published before 30 December 2022. The search terms used in the databases are listed in **Supplementary Table S1**.

### Inclusion criteria

The following studies were included:

(1) The subjects were children and adolescents;
(2) Inclusion of observational epidemiological studies, including cohort, nested case–control, and cross-sectional studies;
(3) Studies with PFAS levels as exposure factors in children/adolescents and the variables related to glucolipid metabolism (glucose, insulin, HOMA-IR, triglyceride (TG), TC, HDL, LDL) were outcomes, and the relationships between them were discussed;
(4) Data such as beta values and 95% confidence intervals (CI) of the correlation between PFAS and outcome variables or convertible related statistics were provided;
(5) When there was overlap between different study populations, the study with the largest sample size and a wider age range was selected.

The following studies were excluded:

(1) The types of studies are reviews, conference abstracts, meta-analyses, lecture papers, and conference reports;
(2) The study subjects are non-natural populations (animals, plants, adults, pregnant women, or other specific populations);
(3) Studies that examined the relationship between PFAS concentrations and other metabolisms among children/adolescents;
(4) Studies that explored the relationship between other environmental exposures and glucolipid metabolism in children/adolescents;
(5) The study designs were flawed, and the quality of the studies was poor.

### Study selection and data extraction

First, we exported the studies in the database using Endnote X9 to automatically detect duplicates and remove them in bulk. Second, we reviewed the titles, abstracts and screened studies that met the inclusion criteria. Additionally, we read the full text, screened studies that met the requirements, and identified studies for inclusion in the meta-analysis. We extracted basic information and available data from these studies. We screened data from the selected studies and extracted the characteristics of each study, including the first author’s surname, year of publication, type of study, number of subjects, covariates, age at exposure to child/adolescent PFAS, type of PFAS, sample type and outcome unit metrics, effect size, and 95%CI.

### Quality assessment

We independently assessed the quality of each study according to the Newcastle–Ottawa Scale (NOS) (18). The NOS consists of three modules with eight items: population selection, comparability, and exposure/outcome. In addition, NOS uses the semi-quantitative principle of the star system to evaluate the quality of literature. Except for comparability, the other items could be rated up to 1 star, with a full score of 9 stars.

### Statistical analysis

Relevant information was extracted from the included studies and a database was established. Stata 15.0 software was used in the meta-analysis to merge the effect values. The adjusted confounders for *β* and 95%CI of the five PFAS (PFOS, PFOA, PFNA, PFHxS, and PFDA) associated with glucolipid metabolism were extracted. As there are few studies on PFDA and glycometabolism, four other PFAS were selected to study the relationship between PFDA and glucose metabolism. The steps were as follows: (1) Data normalization: The relevant effect values extracted from the studies were normalized to exposures in increasing units of 10 log- ng/ml, while glucose, insulin, HOMA-IR, TC, TG, HDL, and LDL were retained in the original units. (2) Heterogeneity test: Heterogeneity between studies was evaluated using the *Q* statistic and *I^2^*. Corresponding to the *Q* statistic, *P >*0.05 or *I^2^* <50% indicated that the heterogeneity among studies was acceptable, and the fixed effect model was chosen to calculate the combined effect values. Otherwise, the heterogeneity of the study was large, and the random-effect model was selected. The forest plot shows the pooled effect values between studies. (3) Subgroup analysis: Subgroup analyses were performed according to the age of children and adolescents divided into children (3–12 years) and adolescent groups (12–19 years), and according to gender divided into male and female groups, and *β* and 95%CI were calculated for each group separately. (4) Sensitivity analysis: Sensitivity analysis was used to evaluate the reliability and robustness of the meta-analysis results, which were evaluated by eliminating studies individually and then pooling them. (5) Publication bias tests: Begg’s and Egger’s tests were used to evaluate the publication bias of the included literature data.

## Results

### Study search and characteristics overview

**Figure 1:**
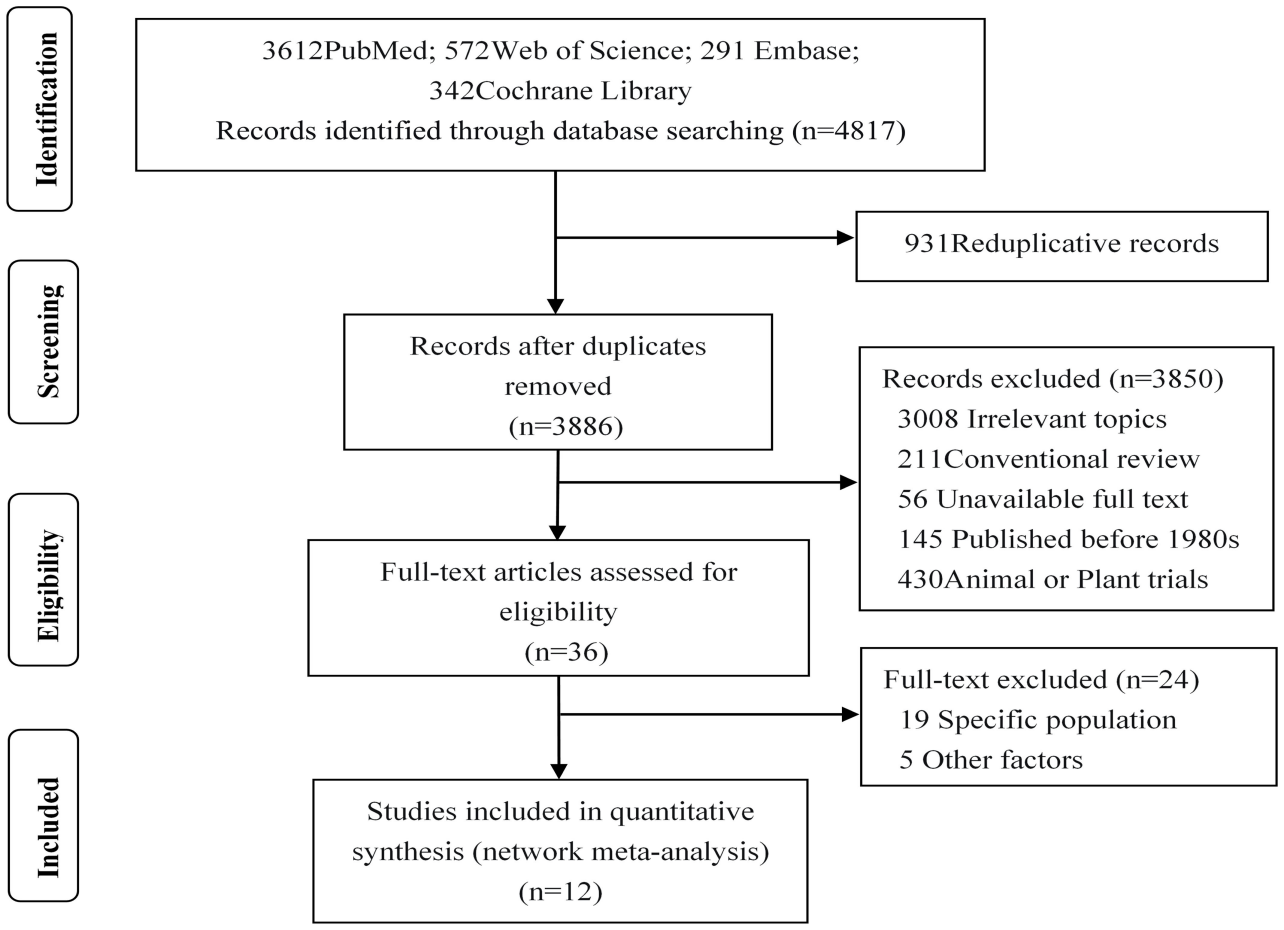
Flow diagram of literature screening.

A total of 4,817 studies were initially included, with 3,886 remaining after excluding duplicates. After reading the titles and abstracts, 3,364 studies were excluded due to unrelated exposures or outcomes, published before the 1980s. We excluded 486 studies in which subjects were animals or plants, and studies in which the exposure-outcome report lacked *β* and 95%CI. In addition, we read the full text of the remaining 36 studies: 19 were excluded because they included pregnant women or adults, two were further excluded due to non-four-spacing categorical variables of exposure factors, another two were excluded due to a lack of original data tables, one was not considered due to the presence of dietary interference factors, and finally 12 studies (19–30) were included in our analysis, as shown in **Figure 1**.

The characteristics of the study are presented in **Supplementary Table S1**. The sample size for inclusion ranged from 40 to 9,462 participants, and the total number of participants in the meta-analysis was 14,364. The ages of the included subjects ranged from 3 to 19 years. Eligible studies were conducted in the United States, Italy, Spain, Antarctica, and China. All studies collected blood samples from children or adolescents, of which three used plasma samples and nine used serum samples, and liquid chromatography-tandem mass spectrometry was used to assess PFAS exposure. The PFAS examined included PFOA, PFOS, PFNA, PFHxS, and PFDA. Glycometabolism indices included fasting blood glucose, fasting insulin, and HOMA-IR. Lipid metabolism indices included TG, TC, HDL, and LDL.

### Associations between PFAS levels and lipid metabolism indices

**Figure 2:**
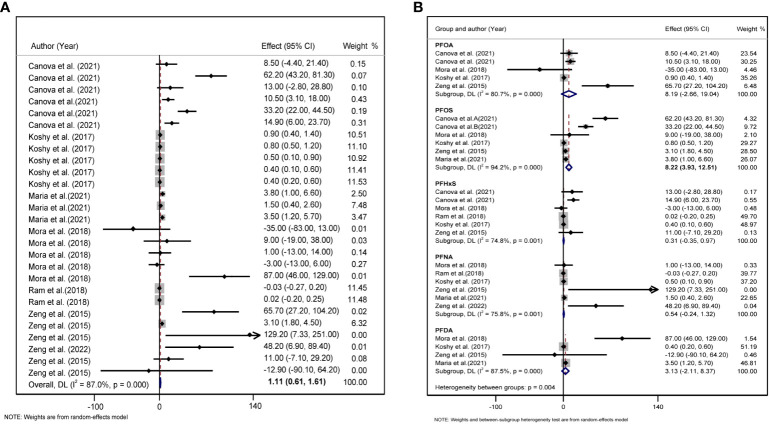
**(A)** Forest plot of PFAS exposure [per 10 log- (ng/ml) increase]and TC. **(B)** Forest plot of each PFAS exposure [per 10 log- (ng/ml) increase] and TC. The X-axis represents the range of combined effect sizes for the meta-analysis, which were set based on the effect size and 95%CI. The points represent the study-specific beta and the horizontal lines correspond to the 95%CI. The gray boxes represent the size of the weight for each study. Pooled β values and 95%CI are presented as diamonds. The vertical dashed line represents the pooled beta efficiency value. The arrows indicate that the 95%CI of the study was outside the range between the two points on the X-axis.

We found a significant positive correlation between PFAS, TC, and LDL levels. In the six studies on the effect of PFAS on TC, the forest plot showed *I^2^ = *87.0%, *P* <0.001, indicating significant heterogeneity among the included studies (**Figure 2A**); therefore, a random-effects model was used to combine the effect values. The results showed an increase in PFAS [per 10 log- (ng/ml)] was positively associated with TC (*β* = 1.110, 95%CI: 0.601, 1.610). We further analyzed the relationship between five different PFAS species and TC, which revealed a positive correlation between PFOS [per 10 log- (ng/ml) increase] and TC (*β* = 8.22, 95%CI: 3.93, 12.51) (**Figure 2B**).

**Figure 3:**
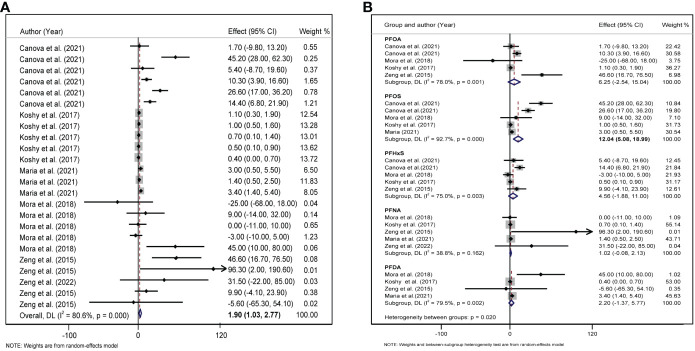
**(A)** Forest plot of PFAS exposure [per 10 log- (ng/ml) increase] and LDL. **(B)** Forest plot of each PFAS exposure [per 10 log- (ng/ml) increase] and LDL. The X-axis represents the range of combined effect sizes for the meta-analysis, which were set based on the effect size and 95%CI. The points represent the study- specific beta and the horizontal lines correspond to 95%CI. The gray box represents the size of the weights for each study. The pooled β and 95%CI are presented as the diamonds. The vertical dashed line represents the pooled beta efficiency value. Arrows indicate that the 95%CI of the study is outside the range between the two points on the X-axis.

A total of five studies were included to explore the combined effect size of the relationship between PFAS and LDL levels. Heterogeneity analysis revealed that. The random-effects model showed that the increase in PFAS [per 10 log- (ng/ml)] was positively correlated with changes in LDL (*β* = 1.90, 95%CI: 1.03, 2.77, *I^2^ = *80.6%, *P* for heterogeneity <0.001) (**Figure 3A**). In our meta-analysis, according to the type of PFAS, only PFOS was positively associated with LDL (*β* = 12.04, 95%CI: 5.08, 18.99) (**Figure 3B**).

We selected 6 and 7 studies to elucidate the relationship between PFAS and TG and HDL, respectively. As shown in **Table 1**, the *I^2^* of included studies were 78.2% and 87%, indicating that there was significant heterogeneity in all studies. Therefore, a random-effects model was used to combine effect values and show forest plots, and the results showed that the PFAS were not significantly correlated with TG and HDL after combining effect values.

**Table 1:** Meta-analysis of the effects of PFAS [per 10 log- (ng/ml) increase] expose on glucolipid metabolism in children and adolescence.

Outcome	Number of studies	Beta (95% CI)	Heterogeneity	
			*P*-value	*I^2^*%
Glucose	4	0.140 (−0.400, 0.680)	0.852	<0.001
Insulin	4	−0.029 (−0.099, 0.042)	0.054	41.2
HOMA-IR	6	−**0.130 (**−**0.200,** −0.059) ^*^	0.004	47.9
TG	6	0.120 (−0.100, 0.340)	<0.001	78.2
TC	6	1.110 (0.601, 1.610) ^*^	<0.001	87.0
HDL	7	−0.469 (−0.031, 0.970)	<0.001	73.5
LDL	5	1.900 (1.030, 2.770) ^*^	<0.001	80.6

*Statistically significant differences. Bold content means significant statistical significance.

### Associations between PFAS levels and glycometabolism indices

**Figure 4:**
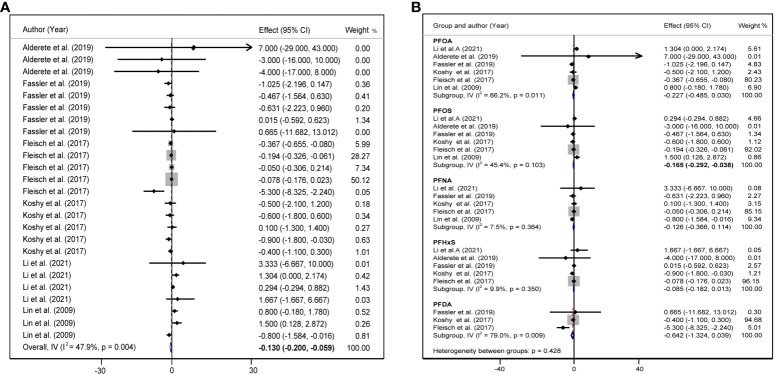
**(A)** Forest plot of PFAS exposure [per 10 log- (ng/ml) increase] and HOMA-IR. **(B)** Forest plot of each PFAS exposure [per 10 log- (ng/ml) increase] and HOMA-IR. The X-axis represents the range of combined effect sizes for the meta-analysis, which were set based on the effect size and 95%CI. The points represent the study-specific beta and the horizontal lines correspond to the 95%CI. The gray box represents the size of the weights for each study. The pooled β and 95%CI are presented as the diamonds. The vertical dashed line represents the pooled beta efficiency value. Arrows indicate that the 95%CI of the study is outside the range between the two points on the X-axis.

We included six studies on the association between PFAS and HOMA-IR, and the meta-analysis showed that childhood exposure to PFAS was weakly but significantly negatively correlated with HOMA-IR (*β* = −0.130, 95%CI: −0.200, −0.059) (**Table 1**, **Figure 4A**). In the six studies on the effect of PFOS on HOMA-IR, the forest plot showed *I^2^ = *45.4%, *P* = 0.103, indicating acceptable heterogeneity among the included studies (**Figure 4B**); therefore, the fixed-effects model was used to combine the effect values. The results showed that an increase in PFOS [per 10 log- (ng/ml)] was negatively associated with HOMA-IR (*β* = −0.165, 95%CI: −0.292, −0.038). In addition, we did not find any significant effects of PFAS on blood glucose and insulin levels (**Table 1**).

### Subgroup analysis

**Figure 5:**
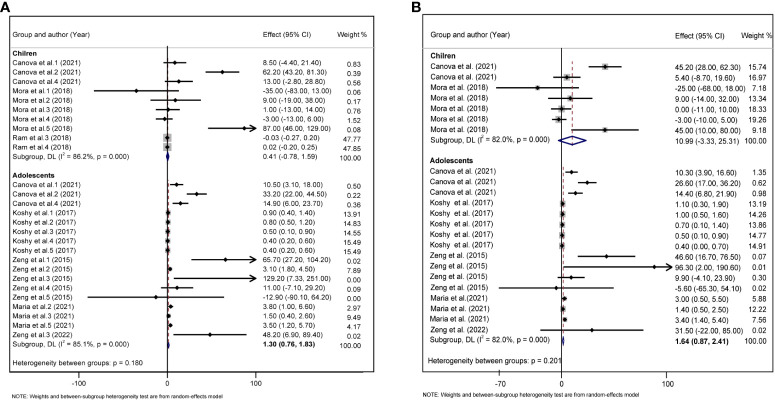
**(A)** Forest plot of PFAS exposure [per 10 log- (ng/ml) increase] and TC during childhood and adolescence. **(B)** Forest plot of PFAS exposure [per 10 log- (ng/ml) increase] and LDL during childhood and adolescence. The X-axis represents the range of combined effect sizes for the meta-analysis, which were set based on the effect size and 95%CI. The points represent the study- specific beta and the horizontal lines correspond to 95%CI. The gray box represents the size of the weights for each study. The pooled β and 95%CI are presented as the diamonds. The vertical dashed line represents the pooled beta efficiency value. Arrows indicate that the 95%CI of the study is outside the range between the two points on the X-axis.

**Figure 6:**
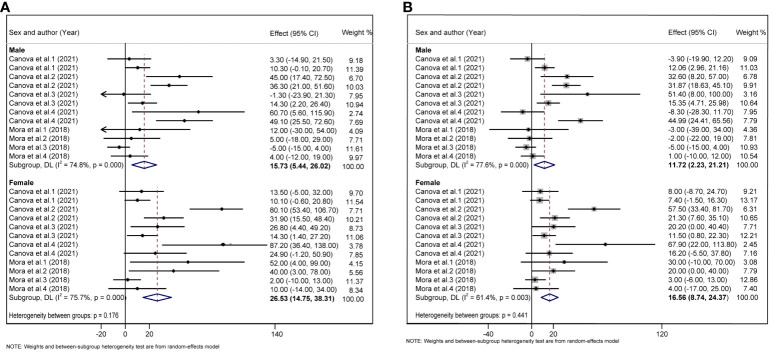
**(A)** Forest plot of PFAS exposure [per 10 log- (ng/ml) increase] and TC in boys and girls. **(B)** Forest plot of PFAS exposure [per 10 log- (ng/ml) increase] and LDL in boys and girls. The X-axis represents the range of combined effect sizes for the meta-analysis, which were set based on the effect size and 95%CI. The points represent the study- specific beta and the horizontal lines correspond to 95%CI. The gray box represents the size of the weights for each study. The pooled β and 95%CI are presented as the diamonds. The vertical dashed line represents the pooled beta efficiency value. Arrows indicate that the 95%CI of the study is outside the range between the two points on the X-axis.

Considering that children are growing and developing, their age, sex, overweight/obesity, and other factors may affect glycolipid metabolism. We divided the study subjects into child and adolescent groups, and male and female groups according to their age and sex, and further performed a subgroup analysis. In adolescents, each 10 log- ng/mL increase in PFAS was associated with an increase of 1.30 units TC (95%CI: 0.76, 1.83) (**Figure 5A**), and each 10 log- ng/mL increase in PFAS was associated with an increased 1.64 units LDL (95%CI: 0.87, 2.41) (**Figure 5B**), but not statistically significant in the children group. In addition, we did not find a significant correlation between PFAS and HOMA-IR in the child and adolescent groups (**Supplementary Figure S2**). PFAS exposure had a positive effect on TC (*β* = 26.53, 95%CI:14.75, 38.31) and LDL (*β* = 16.56, 95%CI: 8.74, 24.37) levels in girls than in boys (**Figures 6A, B**).

### Sensitivity analysis and publication bias

We conducted a sensitivity analysis through the elimination method one by one and found that the combined effect size and 95%CI did not change significantly, indicating that our study was stable and reliable (**Supplementary Figures S1–S7**). Begg’s and Egger’s tests were used to examine the publication bias in the included studies. The results showed no significant asymmetry in the funnel plots of the included studies on glucose, insulin, HOMA-IR, TG, and HDL levels, suggesting that no significant publication bias was found. (**Supplementary Table S3**).

## Discussion

PFAS are widely used in many industries because of their stable physical and chemical properties, and they hardly decompose in nature, which means that they exist in the environment for a long time. The National Academies of Sciences, Engineering, and Medicine (NASEM) determined that the potential adverse effects may occur when PFAS serum levels in the body range from 2 ng/ml to 20 ng/ml, especially in susceptible individuals, and if PFAS serum levels in the body exceed 20 ng/ml, the risk of adverse effects increases (31). Relevant data show that the main exposure route of PFAS in the body is through drinking contaminated water or eating fish in some highly exposed people in special areas and occupational exposure (such as in the production process of fluorine compounds) in some factory workers. The primary sources of exposure to PFAS and their precursors appear to be food and food packaging and consumer products, particularly non-polymer aftermarket treatments (32). Compared with adults, children’s metabolic systems are less mature, but more vulnerable to environmental and other hazards. Simultaneously, adolescents are more sensitive to the environment due to puberty, which might be influenced by hormonal levels. However, the relationship between PFAS and glucolipid metabolism in children remains to be elucidated.

In the present study, we conducted a systematic review and meta-analysis to evaluate the relationship between PFAS and indicators of glucolipid metabolism. We searched four major databases and included 12 studies with a total of 14,364 children or adolescents. The results showed that PFAS were weakly negatively correlated with HOMA-IR, and positively correlated with TC and LDL levels.

Many studies have examined the relationship between PFAS and lipids in humans, and a meta-analysis that included 29 adult PFAS exposure and lipids showed that PFOA and PFOS were positively associated with TC and LDL (33). In children and adolescents, the evidence is also fairly consistent, suggesting a positive association between PFAS, TC, and LDL, and the results of our meta-analysis further support this evidence. Among the five PFAS, PFOS may be the key compound determining the effect of PFAS on lipids, which is also true for glycolytic metabolism indicators. This may be due to the hydrophobic and lipophobic nature of PFOS, which is widely used in life production-related applications (34). However, it has also been shown that PFOA and PFNA exposure and TC and LDL levels in children and adolescents are positively correlated, although the overall effect of our meta-analysis was not significant. The effect of PFAS exposure on TC and LDL was positively correlated only in the adolescent group, probably because adolescents are more likely to ingest exogenous PFAS and have greater bioaccumulation than younger children. The positive correlation was more sensitive in girls relative than in boys, and more studies are needed in the future to investigate the relationship between PFAS and lipids in different populations. Obesity can also affect lipid metabolism index in children. Obese children can be exposed to more PFAS through diet and food packaging, or PFAS are more likely to cause abnormal glucolipid metabolism in the obese state. Among the included articles, only one study focused on overweight and obese children (21), which showed that higher PFAS exposure was associated with increased 2-hour glucose levels. Another study by Timmermann et al. showed a positive association between PFOA and PFOS with TG, HOMA-IR, insulin, and glucose in overweight children, while there was no statistical difference in the group of (n = 59) normal children (n = 405) (35). This suggests that overweight/obesity may be an important factor influencing the relationship between PFAS and glucolipid metabolism; however, this has not been considered in many studies. Future population studies need to consider the unique growth and developmental characteristics of children, such as overweight/obesity, to interpret the relationship more rigorously between PFAS and indicators of glucolipid metabolism in children and adolescents.

In our meta-analysis, exposure to PFAS in children and adolescents was found to be positively associated with insulin resistance. Consistent with our meta-analysis, a Swedish cohort study found that long-term PFAS exposure is associated with lower insulin resistance (14). However, Sweden is a Nordic country with a high fish intake. The effects of PFAS exposure varied across countries. In contrast to our results, a two-fold increase in PFHxS concentration during pregnancy was significantly associated with increased fasting plasma glucose, fasting insulin, and HOMA-IR values (36), which may be caused by specific physiological changes during pregnancy. This would make pregnant women more susceptible to the risk of hyperglycemia, insulin resistance, and even GMD, and PFAS exposure further strengthens the risk of insulin resistance. The same type of PFAS related to lipid metabolism, PFOS was shown to be associated with insulin resistance. This suggests that PFOS may be the most relevant PFAS for glucolipid metabolism in children and adolescents, and has the potential to play a decisive role. In addition, obesity, adolescence, and sex may also affect the insulin resistance index. Except for the study by Fassler et al. (22), which included women, the gender distribution of other studies was consistent. However, as these studies did not fully adjust for sex, we were unable to conduct a stratified analysis. Three articles on inclusion involved subjects in adolescence, which were further divided into children and adolescent groups based on age. The results showed that the correlation between PFAS and HOMA-IR was not statistically significant between the two groups. We suggest that the effect of PFAS on HOMA-IR may be distributed throughout the children–adolescent age group.

At present, the mechanism by which PFAS cause glucolipid metabolism disorders remains unclear, and the conclusions are inconsistent (37). Studies have shown that PFAS can indirectly inhibit tyrosine phosphorylation of insulin receptor substrates (IRSs) to prevent insulin signaling pathways and reduce insulin sensitivity by activating protein kinases. Some researchers also believe that PFAS have structural homology with some fatty acids in the human body, which increases the expression of genes related to fatty acid oxidation and causes oxidative stress in the body, thereby aggravating insulin resistance (38) It has also been suggested that the effects of PFOS/PFOA on cholesterol and lipid homeostasis may be mediated by activation of the peroxisome proliferator activated receptor alpha (PPARα) pathway or inhibition of hepatocyte nuclear factor 4-alpha (HNF4α) protein expression (39, 40). In contrast to human statistics, animal experiments have shown that exposure to PFAS in rats or mice may reduce cholesterol or lipoproteins in the body (41). Nevertheless, some scholars believe that the PFAS accumulation time in rats or mice is less than that in humans (42), and the toxic effects are inconsistent due to different exposure levels and species. In addition, the pathway of PFAS exposure among mice and rats is relatively single, mainly by gavage, while humans can be exposed to PFAS through multiple pathways, such as diet, drinking water, and skin contact, and the exposure factors are more complex. However, compared to rats and mice, some PFAS may accumulate in humans to a much greater extent.

The major strengths of our study are as follows: we focused on the effects of exposure to multiple types of PFAS during childhood and adolescence on glucolipid metabolism. Second, age, sex, and obesity were included in the subgroup analysis, considering the unique physiological characteristics of children. We included not only glucose and insulin as indicators of glucose metabolism but also insulin resistance indices so that the results would be more consistent with the effects of metabolic diseases. In terms of blood lipids, TC, TG, HDL, and LDL were fully included, which could provide a more specific and comprehensive consideration in cases where lipid metabolism indicators are more relevant. However, our results showed that PFOS was most closely associated with glucolipid metabolism, with a significant correlation.

Although we tried to adjust the unit consistency of outcome indicators as much as possible, the effect sizes of some outcome indicators did not change significantly after adjustment; therefore, we retained such effect sizes, and there may be some errors in the results. Children from different regions showed varying concentrations of PFAS exposure due to ethnicity, diet, and lifestyle. The average concentrations of PFOA in children from Italy and Chinese Taiwan were 51.6 ng/ml and 26.2 ng/ml, respectively (19, 27), whereas the average concentrations in the other 10 eligible studies conducted in America and Spain were less than 10 ng/ml, which may have led to the different effects of PFAS on children’s glucose and lipid metabolism in different regions. However, we were unable to conduct a stratified analysis by ethnicity, diet, and region in the included studies. Future investigations could further refine these factors to explore the health benefits of PFAS. Furthermore, owing to the limited number of available studies and inconsistencies in study designs and statistical methods, the findings still need to be viewed with caution and objectivity. In recent years, as people have become increasingly aware of the impact of PFAS on the human body, many countries have introduced relevant policies to restrict the use of PFAS and the emergence of substitutes, which have gradually replaced mainstream PFAS. However, the abuse of PFAS in the past still affects public health, and the effect of substitutes on human health requires further research.

## Conclusions

Because of the impact of PFAS on glucolipid metabolism in children, it is recommended that relevant departments formulate corresponding policies to control and limit the content of PFAS in consumer products. Pediatric clinicians should also be aware of the impact of environmental exposure on children and adolescents and strengthen health education for children.

## Data availability statement

The original contributions presented in the study are included in the article/**Supplementary Material**. Further inquiries can be directed to the corresponding author.

## Author contributions

QZ: Data curation, Formal analysis, Writing – original draft. WY: Funding acquisition, Methodology, Software, Writing – original draft, Writing – review & editing. SG: Resources, Writing – review & editing. XL: Project administration, Supervision, Validation, Writing – review & editing.
