# Supplementary material for: The effect of PFAS exposure on glucolipid metabolism in children and adolescents: a meta-analysis
Source: Front Endocrinol (Lausanne). 2024 Feb 14;15:1261008. doi: 10.3389/fendo.2024.1261008 (PMC10902913; doi:10.3389/fendo.2024.1261008)
Supplement: Supplementary file 1 [file DataSheet_1.docx]

Supplementary Material

**The effect of PFAS exposure on glucolipid metabolism in children and adolescents: a meta-analysis**

Xiaonan Li *, Qingqing Zheng, Wu Yan, Shenghu Gao

*** Correspondence:** Xiaonan Li: xiaonan6189@163.com

# Supplementary Tables and Figures

## Supplementary Tables

**Table S1** Search keywords for meta-analysis of PFAS exposure and glucose metabolism and lipid metabolism

| Search | Search Term |
| --- | --- |
| #1 | “per and polyfluorinated chemicals” OR “polyfluoroalkyl chemicals” OR “perfluorinated chemicals” OR “fluorine compounds” OR “polyfluoroalkyl compounds” OR “fluorinated organic compounds” OR “perfluoroalkyl and polyfluoroalkyl substances” OR “perfluorinated alkyl substances” OR “perfluorooctane sulfonate” OR “perfluoroalkyl sulfonate” OR “perfluorohexane sulfonate” OR “perfluorinated acid” OR “perfluorononanoic acid” OR “perfluorooctanoic acid” OR “perfluorooctane sulfonic acid” OR “perfluorohexane sulfonic acid” OR “perfluoroalkyl acids” OR “alkanesulfonic acids” OR perfluorinated OR fluorine OR halothane OR perfluorooctanoate OR fluorocarbons OR “PFAS” OR “PFOA” OR “PFOS” OR “PFAA” OR “PFNA” OR “PFC” OR “PFHxS” OR “PFOSA” |
| #2 | glucose OR “blood sugar” OR “blood glucose” OR glycemic OR “FPG” OR “FBG” OR “Glu” OR “FBS” OR “glycosylated hemoglobin” OR “glycated hemoglobin” OR “GHB” OR “HbA1c” OR “HBALC” ORinsulin OR hyperinsulinemia OR “insulin resistance” OR “Insulin Regular” OR “Regular Insulin” OR Novolin OR Iletin OR “Soluble Insulin” OR “Insulin Soluble” OR “Insulin A Chain” OR “Insulin B Chain” OR “Chain Insulin B” OR “Sodium Insulin” OR “Insulin Sodium” OR “HOMA IR” OR InS OR FIN OR IRI |
| #3 | “blood lipid” OR “serum lipid” OR “blood fat” OR “lipid profile” OR cholesterol OR cholesterin OR cholestenone OR “CHOL” OR “TC” OR “low density lipoprotein” OR “LDL Cholesterol” OR “LDL C cholesterol” OR “LDL” OR “high density lipoprotein” OR “HDL Cholesterol” OR “HDL Cholesterol” OR “HDL” OR “HDL C” OR Triglyceride OR triacylglycerol OR “TG” OR “TAG” OR “TRIG” OR |
| #4 | #1 AND #2 AND #3 |

**TableS2** Main characteristics of studies included in the meta-analysis

| Country | (Author, year,) | Study design | Population | PFAS | Sample type | Age of exposure/outcome assessment | Outcome | | | Adjustment variables | NOS |
| --- | --- | --- | --- | --- | --- | --- | --- | --- | --- | --- | --- |
| Italy | Canova et al. 2021 | Cross-sectional Study | 9,362 | PFOA PFOS PFHxS PFNA | Serum | Children 8-11 years old  Teenager14-19 years old | | TC TG HDL-C LDL-C BMIz BP | Adjusted for age, gender, country of birth, data on food consumption, degree of physical activity, salt intake, smoking status (for adolescents only), time-lag between the beginning of the study and the date of enrollment. | | 7 |
| America | Li et al. 2021 | Cohort Study | 616 | PFOA PFOS PFHxS PFNA | Serum | Cord-3 years old-8 years old-12 years old | | Glucose Insulin Wasit Cir Adiponectin Leptin HOMA-IR HDL TG BP | | Adjusted for maternal age, maternal education, maternal BMI, maternal smoking, pubertal stage, child age, child race, child sex, breastfeeding | 7 |
| Spain | Alderete et al.2019 | Cohort Study | 40（BMI ≥96.8 ± 3.5%） | PFOA PFOS PFHxS PFNA | Plasma | Children 8-14years old | | Glucose Insulin  2h-Glucose 2h-Insulin HOMA-IR | Adjusted for sex, baseline social position (categorical), baseline outcome as well as baseline and change in age at follow-up, pubertal status (categorical) as well as baseline and change in body fat percent at follow-up. | | 8 |
| America | Fassler et al.2019 | Cross-sectional Study | 353 | PFOA PFOS PFHxS PFNA | Serum | Girls 6-8 years old | | Glucose Insulin HOMA-IR | Adjusted for race/ethnicity and age in month | | 6 |
| America | Mora et al.2018 | Cohort Study | 653 | PFOA PFOS PFHxS PFNA PFDA PFUdA EtFOSAAMeFOSAA | Plasma | Median, 7.7years of age | | TC TG LDL HDL | Adjusted for maternal education, prenatal smoking, and child’s sex, race/ethnicity, age at lipids/ALT measurements, and all PFAS analytes. | | 7 |
| America | Ram.2018 | Cohort Study | 458 | PFASs PFHxS PFNA PFOA PFOS | Serum | Children 6-11 years old | | HDL TC | Adjusted for gender, race/ethnicity, age, age2, poverty income ratio, body mass index percentiles, fasting time, and exposure to second hand smoke. | | 7 |
| America | Koshy et al.2017 | Cross-sectional Study | 308 | PFOA PFOS PFHxS PFNA PFDA PFUdA | Serum | Children were born between 11,9, 1993 and 10,9,2001 | | HOMA-IR TG TC LDL HDL | Adjusted for each column represents an examination of a single exposure variable or study arm controlled for sex, race, caloric intake, physical activity, cotinine concentration and BMI category (except when the outcome examined was BMI); | | 6 |
| America | Fleisch et al.2017 | Cohort Study | 665 | PFOA PFOS PFHxS PFNA PFDeA | Plasma | Median, 7.7 years of age | | HOMA-IR | Adjusted for characteristics of child (age, sex, race/ethnicity), mother (age, education, parity, smoking during pregnancy), neighborhood census tract at enrollment (median household income, percent below poverty), and pregnancy hemodynamics (time of blood draw in weeks gestation) | | 8 |
| China | Zeng et al.2015 | Cross-sectional Study | 225 | PFOA PFOS PFBS PFHxA PFHxS PFNA PFDA PFDoA PFTriDA | Serum | Teenager 12-15 years old | | TG TC HDL LDL | Adjusted for age, gender, BMI, parental education level, exercise and ETS exposure. | | 7 |
| Antarctica | Maria.2021 | Cross-sectional Study | 940 | PFHxS PFHpS PFOS PFOA PFNA PFDA | Serum | 16.4 ± 1.3 years | | TG TC HDL LDL | Adjusted for age, sex, BMI and for lifestyle and diet variables: total cholesterol for intake of junk food (sausages, pizza, hamburger), snacks (chips, biscuits cakes and buns), full fat dairy products, fat and lean candy intake; triglycerides for physical activity, intake of cheese, fish liver and for time since the last meal. lean fish; HDL-cholesterol for chewed tobacco use (snuff), vegetables intake, fish liver intake; apolipoprotein A1 for vegetables and fruits intake, fish liver, snacks and fish; LDL-cholesterol and apolipoprotein B for intake of junk food, full fat dairy products. | | 7 |
| America | Zeng et al.2022 | Cross-sectional Study | 270 | PFNA | Serum | Boys 15-17years old | | TG TC HDL LDL | Adjusted as age, race, poverty-income ratio, body mass index, and serum cotinine levels | | 7 |
| America | Lin et al.2008 | Cross-sectional Study | 474 | PFOA PFOS PFNA PFHxS | Serum | Mean age 15.5 (SEM0.2) years old | | Glucose Insulin HOMA-IR | Model 1 adjusted for age, sex, race, health behaviors (smoking status, alcohol intake, and household income), measurement data (waist circumference, CRP, and insulin/glucose/HOMA) medications. | | 7 |

**Table S3:** Begg's and Egger's test to test the publication bias

| Outcomes | Number of Studies | Begg’s test | | Egger’s test | |
| --- | --- | --- | --- | --- | --- |
|  |  | z | *P*-values | t | *P*-values |
| Glucose | 4 | 0.660 | 0.511 | -0.710 | 0.494 |
| Insulin | 4 | 0.220 | 0.827 | 1.080 | 0.301 |
| HOMA-IR | 6 | 0.120 | 0.907 | -0.290 | 0.774 |
| TG | 6 | 1.820 | 0.068 | 1.900 | 0.069 |
| TC | 6 | 0.940 | 0.348 | 4.980 | ＜0.001 |
| HDL | 7 | 0.050 | 0.959 | 1.210 | 0.237 |
| LDL | 5 | 1.020 | 0.309 | 4.440 | ＜0.001 |

## Supplementary Figures


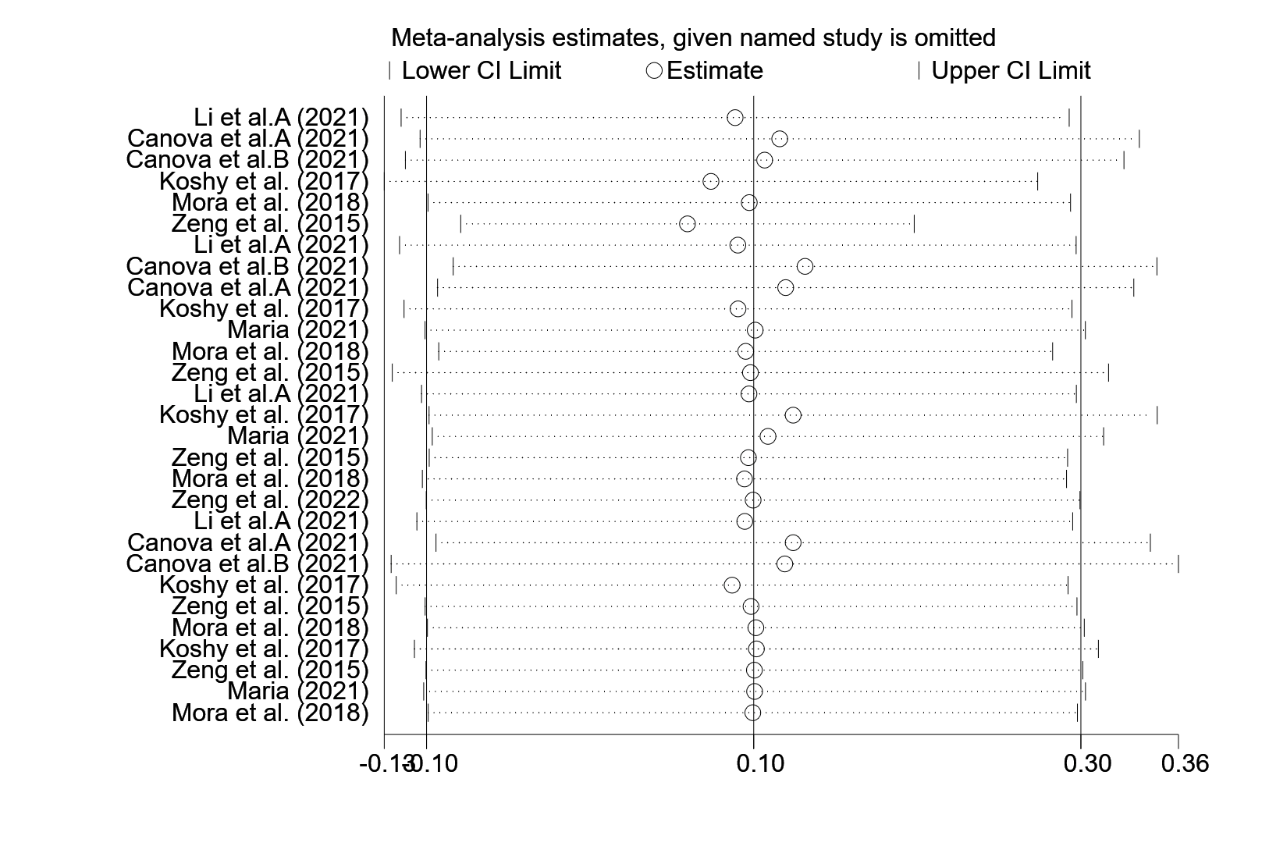


**Figure S1** Sensitivity analysis of PFAS and TG


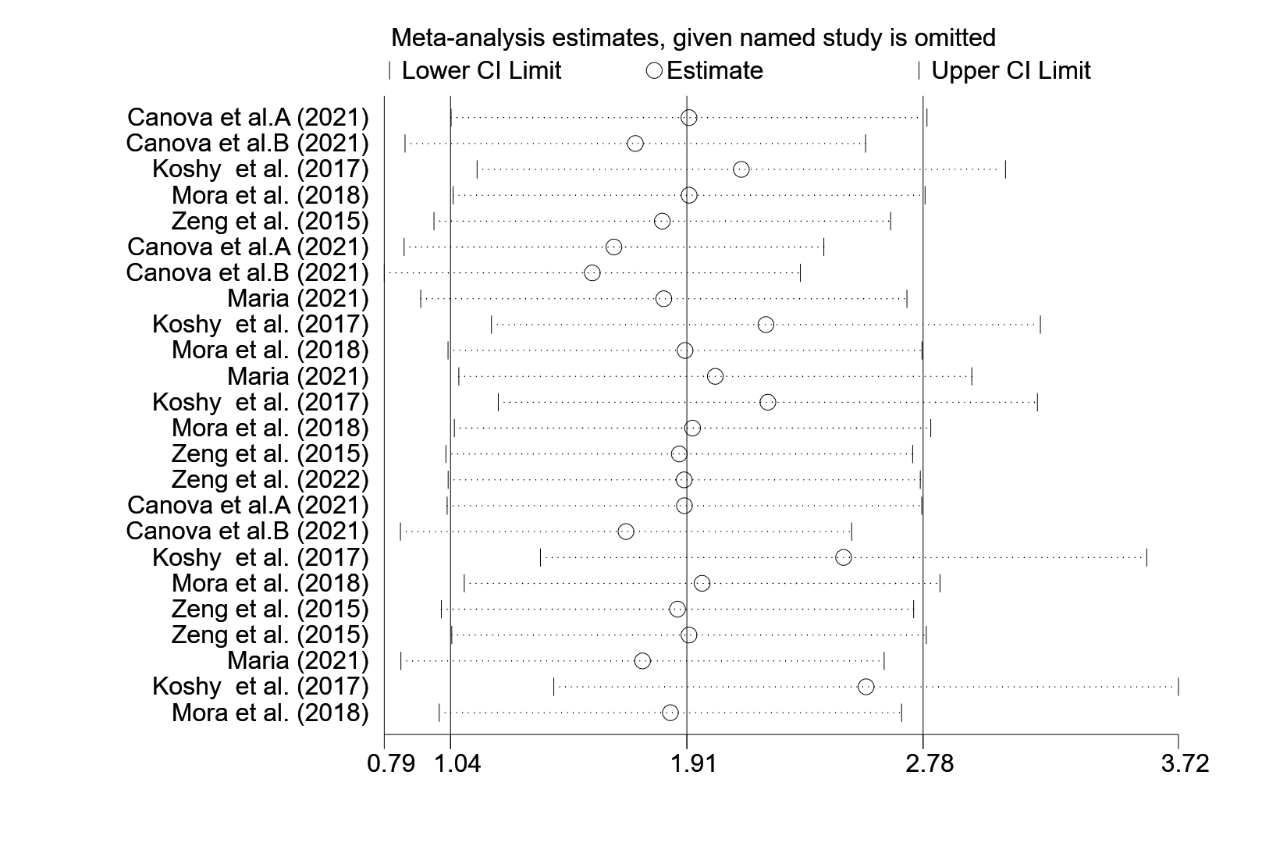


**Figure S2** Sensitivity analysis of PFAS and LDL


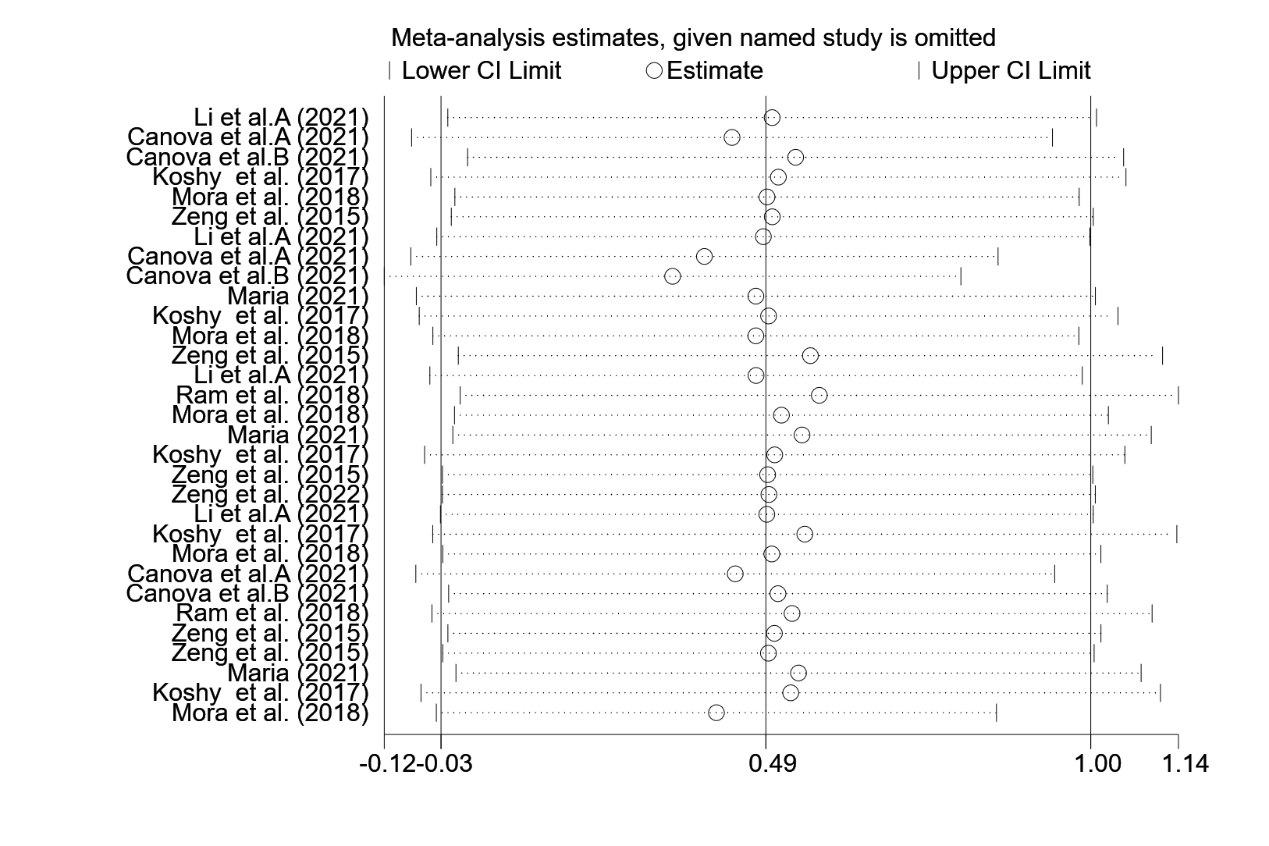


**Figure S3** Sensitivity analysis of PFAS and HDL


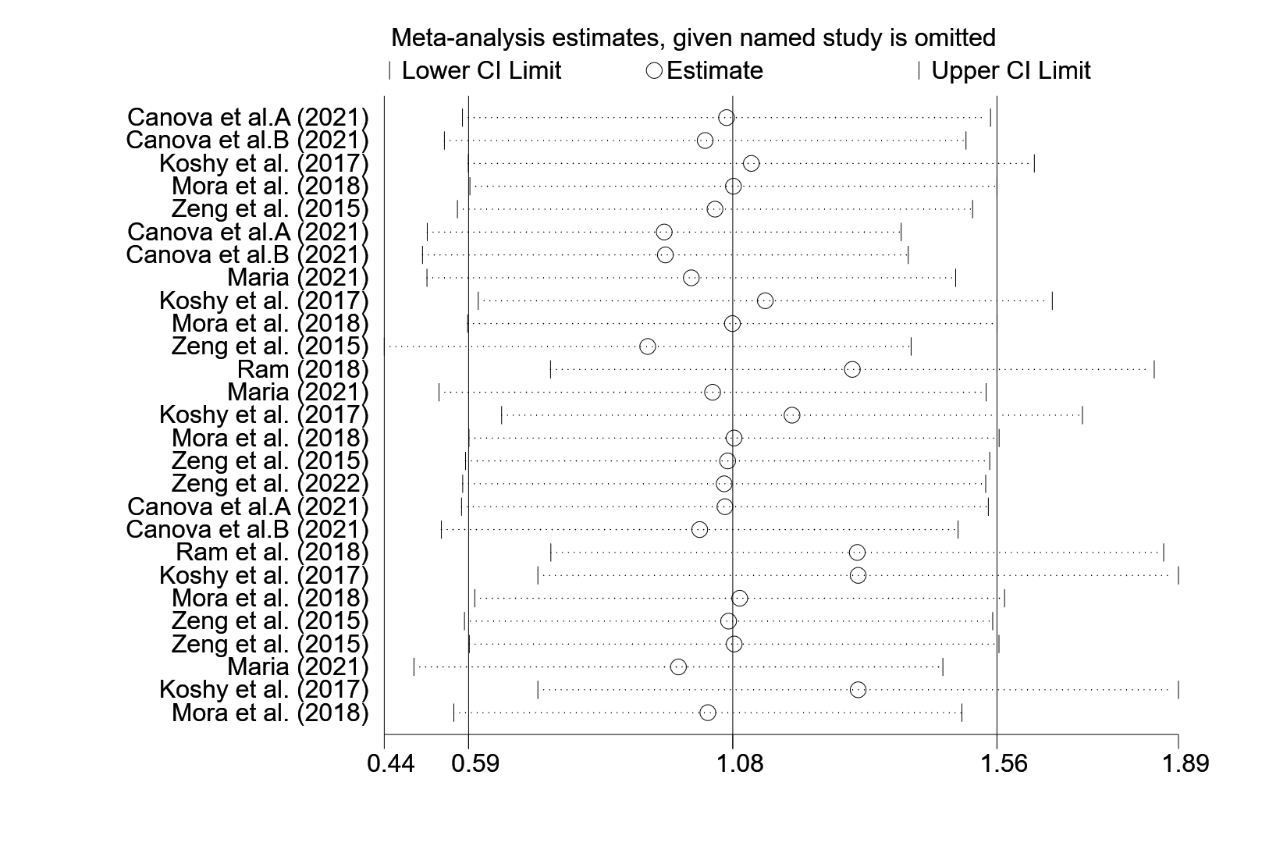


**Figure S4** Sensitivity analysis of PFAS and TC


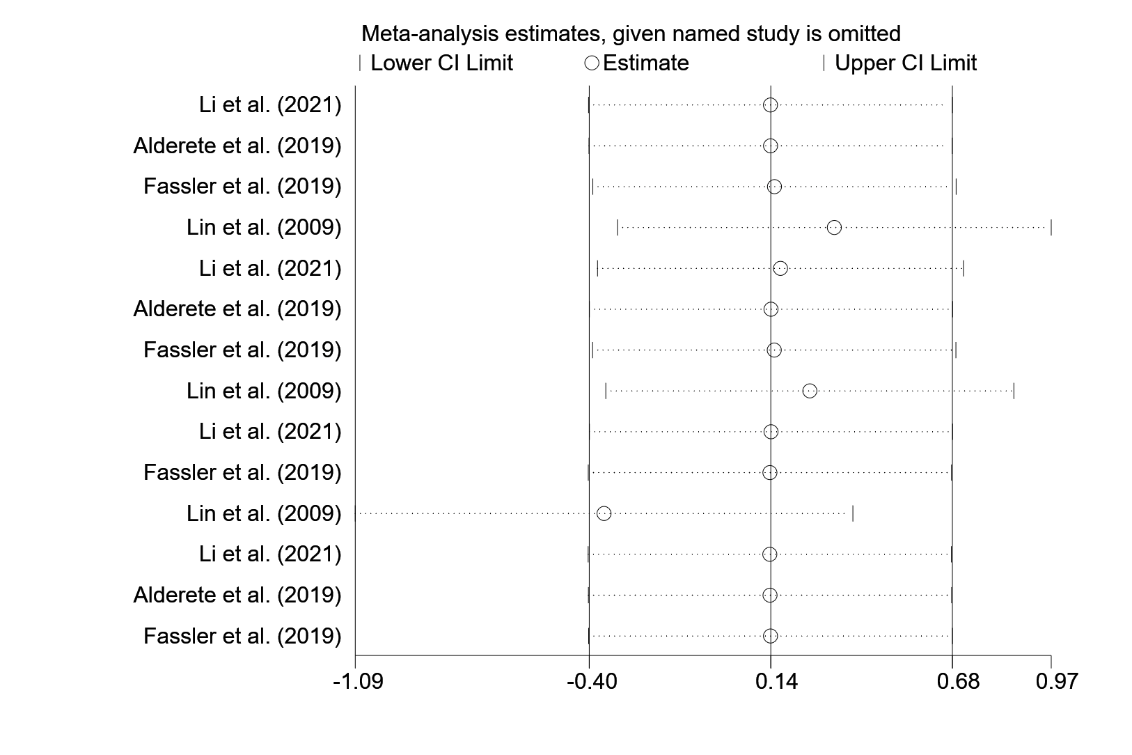


**Figure S5** Sensitivity analysis of PFAS and glucose


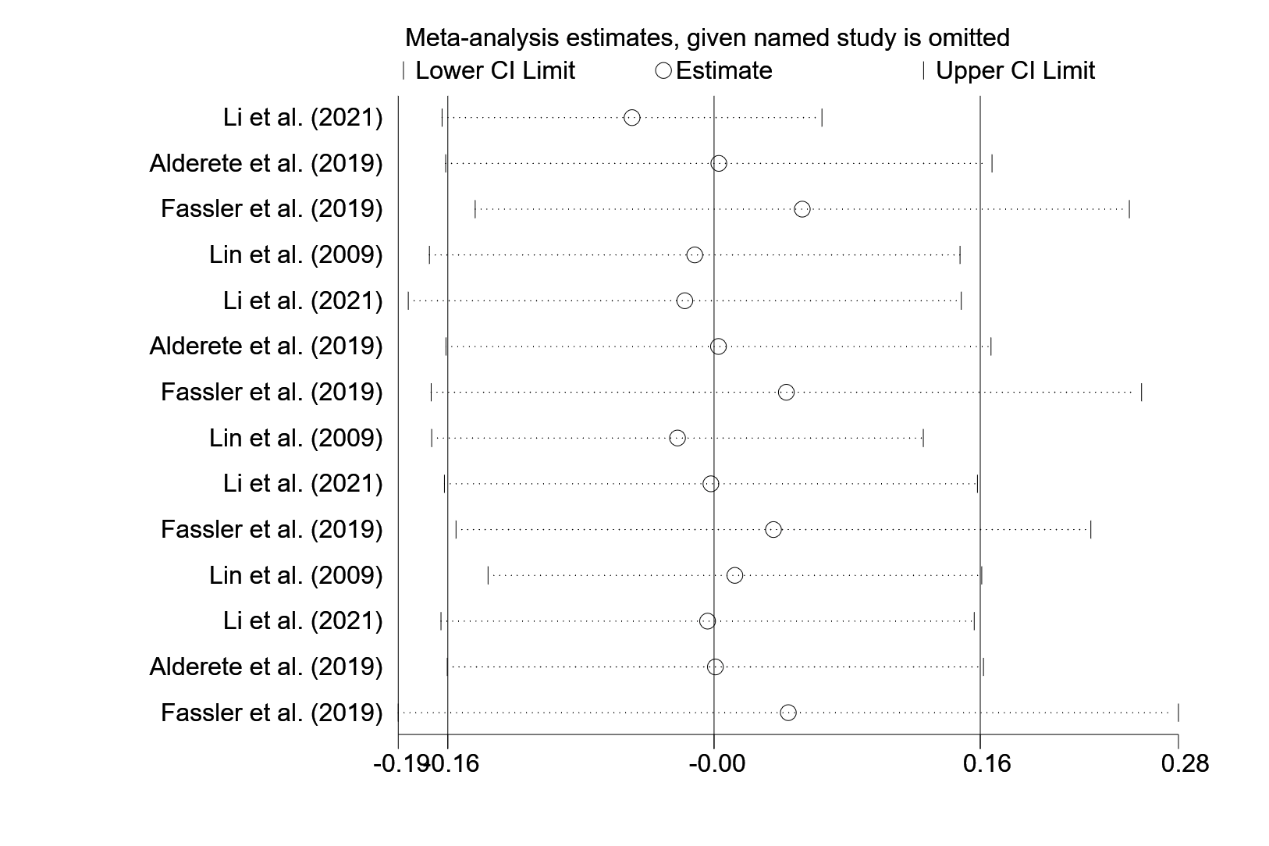


**Figure S6** Sensitivity analysis of PFAS and insulin


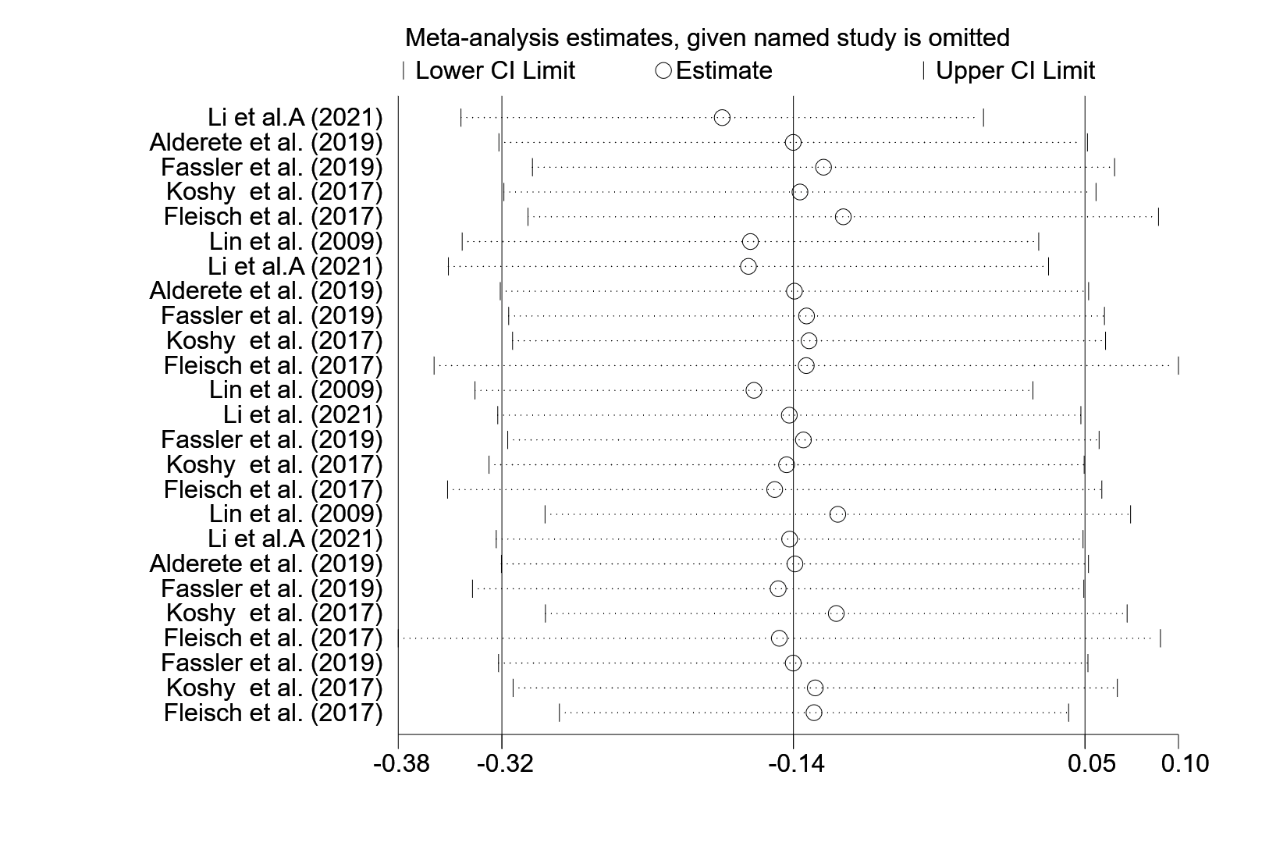


**Figure S7** Sensitivity analysis of PFAS and HOMA-IR
